# Treatment outcomes for newly diagnosed, treatment-naïve *TP53*-mutated acute myeloid leukemia: a systematic review and meta-analysis

**DOI:** 10.1186/s13045-023-01417-5

**Published:** 2023-03-06

**Authors:** Naval G. Daver, Shahed Iqbal, Camille Renard, Rebecca J. Chan, Ken Hasegawa, Hao Hu, Preston Tse, Jiajun Yan, Michael J. Zoratti, Feng Xie, Giridharan Ramsingh

**Affiliations:** 1 University of Texas MD Anderson Cancer Center, 1515 Holcombe Blvd, Houston, TX 77030 USA; 2 Gilead Sciences, Inc, 333 Lakeside Dr, Foster City, CA 94404 USA; 3 McMaster University, 1280 Main St W, Hamilton, ON L8S 4L8 Canada

**Keywords:** Acute myeloid leukemia, *TP53* mutations, Intensive chemotherapy, Hypomethylating agents, Venetoclax

## Abstract

**Background:**

*TP53* mutations, which are present in 5% to 10% of patients with acute myeloid leukemia (AML), are associated with treatment resistance and poor outcomes. First-line therapies for *TP53*-mutated (*TP53*m) AML consist of intensive chemotherapy (IC), hypomethylating agents (HMA), or venetoclax combined with HMA (VEN + HMA).

**Methods:**

We conducted a systematic review and meta-analysis to describe and compare treatment outcomes in newly diagnosed treatment-naïve patients with *TP53*m AML. Randomized controlled trials, single-arm trials, prospective observational studies, and retrospective studies were included that reported on complete remission (CR), CR with incomplete hematologic recovery (CRi), overall survival (OS), event-free survival (EFS), duration of response (DoR), and overall response rate (ORR) among patients with *TP53*m AML receiving first-line treatment with IC, HMA, or VEN + HMA.

**Results:**

Searches of EMBASE and MEDLINE identified 3006 abstracts, and 17 publications describing 12 studies met the inclusion criteria. Random-effects models were used to pool response rates, and time-related outcomes were analyzed with the median of medians method. IC was associated with the greatest CR rate of 43%, and CR rates were 33% for VEN + HMA and 13% for HMA. Rates of CR/CRi were comparable for IC (46%) and VEN + HMA (49%) but were lower for HMA (13%). Median OS was uniformly poor across treatments: IC, 6.5 months; VEN + HMA, 6.2 months; and HMA, 6.1 months. For IC, the EFS estimate was 3.7 months; EFS was not reported for VEN + HMA or HMA. The ORR was 41% for IC, 65% for VEN + HMA, and 47% for HMA. DoR was 3.5 months for IC, 5.0 months for VEN + HMA, and was not reported for HMA.

**Conclusions:**

Despite improved responses seen with IC and VEN + HMA compared to HMA, survival was uniformly poor, and clinical benefits were limited across all treatments for patients with newly diagnosed, treatment-naïve *TP53*m AML, demonstrating a significant need for improved treatment for this difficult-to-treat population.

**Supplementary Information:**

The online version contains supplementary material available at 10.1186/s13045-023-01417-5.

## Background

Acute myeloid leukemia (AML) is a hematologic malignancy that develops from clonal expansion of myeloid precursors residing in the bone marrow [1]. In patients with AML, leukemic blasts infiltrate the bone marrow and disrupt normal hematopoiesis. AML typically occurs in adults aged more than 45 years; the median age at diagnosis is 68 years. In 2021, there were an estimated 20,240 new cases of AML in the United States [2]. Based on the latest available data (2011–2017), the estimated 5-year survival rate of all patients with AML was 29.5%. A mainstay of AML treatment has been the combination of cytarabine and an anthracycline, e.g., “7 + 3” and “FLAG-Ida” (fludarabine, cytarabine, idarubicin, and granulocyte colony stimulating factor) regimens as intensive induction chemotherapy [3, 4]. For patients unable to tolerate intensive chemotherapy (IC), hypomethylating agents (HMA) are frequently used to treat AML. Since the approval of the oral BCL-2 inhibitor venetoclax (VEN) [5], which demonstrated improved response rates in the Phase 3 registration study, VIALE-A [6], an HMA (e.g., azacitidine [AZA] or decitabine [DEC]) is frequently combined with VEN for the frontline treatment of AML, particularly for patients aged 75 or more years or those unable to tolerate IC [6].

A subset of the AML patient population is characterized by mutations in the *TP53* gene, which encodes a transcription factor (p53) that serves as a critical tumor suppressor [7]. Stress, such as damage to DNA, activation of oncogenes, and depletion of ribonucleotides, triggers activation of p53, which then regulates expression of various genes required for DNA repair, cell differentiation, cell cycle arrest, and apoptosis. Most mutations are missense alterations that occur in the DNA-binding domain and often result in decreased or absent DNA binding [7, 8], but in some cases, the mutations can result in a mutated p53 that exerts dominant-negative influence on residual wild-type p53 [8]. Mutated *TP53* (*TP53*m) has been detected in 5–15% of patients with de novo AML [9, 10] and 17.6% of those with secondary AML [11]. Among patients with therapy-related AML, this mutation is detected in about 30% of cases [12]. In addition, *TP53*m is associated with low blast counts, complex karyotypes, and underrepresentation of concurrent *FLT3*, *RAS*, *NPM1*, and *RUNX1* mutations [9].

AML patients with *TP53*m have significantly poorer prognosis and lower overall survival (6.5 vs. 33.6 months) compared to *TP53* wild-type AML patients due to resistance to standard AML therapies [13]; worse outcomes have been reported for *TP53m* patients compared to *TP53* wild-type patients following treatment with IC or low-intensity chemotherapy [9]. Even with recently approved HMA + VEN–based therapies, the median overall survival (OS) remains low at only 5 to 6 months, despite encouraging complete remission (CR)/incomplete hematologic recovery (CRi) rates of 40–60% [14–16]. Optimal treatment for the subpopulation of patients with *TP53*m AML has not been established. A thorough understanding of therapy-specific clinical outcomes over the past several decades may help elucidate the magnitude of unmet therapeutic need in this patient population and establish historic expectations for novel therapies and combinations being developed in this space. This systematic review and meta-analysis aims to evaluate outcomes associated with IC, HMA, and VEN + HMA in newly diagnosed, treatment-naïve *TP53*m AML.

## Methods

This systematic literature review and meta-analysis was conducted in accordance with Preferred Reporting Items for Systematic Reviews and Meta-Analyses (PRISMA) guidelines [17].

### Search strategy

EMBASE and MEDLINE were searched from their inception through May 20, 2021. Search terms were specific to the population of interest, the types of treatment interventions, and the types of study design. Search results were limited to human studies and English language. Details of the search strategy are provided in Additional File 1: Table S1. Studies of newly diagnosed or treatment-naïve patients with *TP53*m AML who received IC, HMA, or VEN + HMA were included. Detailed eligibility criteria, which were defined according to the Population, Interventions, Comparisons, Outcomes, and Study design (PICOS) statement [18], are summarized in Table 1. In addition, studies had to be randomized controlled trials (RCTs), single-arm or nonrandomized clinical trials, or prospective or retrospective observational studies with at least 20 patients included. In cases where selected study arms or patient subgroups within a study were eligible, the publication was included in the analysis. Study design, patient characteristics, and outcomes, where available, were extracted.

**Table 1:** Eligibility criteria based on Population, Interventions, Comparisons, Outcomes, and Study design criteria

Component	Criteria
Population	Patients with acute myeloid leukemia with mutated *TP53* receiving first-line treatments
Intervention	Intensive chemotherapy Hypomethylating agents as monotherapy Venetoclax + hypomethylating agents
Outcomes	Primary outcomes: Complete remission (CR) CR with incomplete hematologic recovery (CRi) Any CR (CR/CRi) Median overall survival
	Secondary outcomes, if available: Event-free survival Duration of response Overall response rate
Study design	Randomized controlled trials Single-arm trials Prospective observational studies with n ≥ 20 Retrospective studies with n ≥ 20

### Study selection and data extraction

Two investigators, working independently and in duplicate, screened all titles and abstracts identified in the initial search against the aforementioned preset eligibility criteria for this analysis. Full-text publications of the studies that fulfilled the criteria in the title/abstract phase were retrieved, and the same 2 investigators assessed the eligibility of each study based on its corresponding full-text publication(s). Within the publication-eligibility assessment process, relevant systematic reviews were identified and were reviewed to cross-reference the search strategy and to identify any missed publications. Discrepancies between the investigators were resolved by discussion. If agreement could not be reached, a third reviewer provided arbitration.

A study-mapping exercise was conducted to match publications reporting on the same study. By using registration numbers, study authors, and sample sizes, the use of the study-mapping exercise enabled us to avoid double counting of outcomes in the final data set. This process ensured that reported outcomes from studies were from distinct patients.

The 2 investigators extracted data from the included studies independently and in duplicate. Study characteristics, patient characteristics, and treatment outcomes were identified, and discrepancies in the 2 investigators’ findings were resolved by discussion.

### Therapy selection

All studies included patients receiving IC, VEN + HMA, and/or HMA alone, but treatment dosage, frequency, and duration varied between studies (these details are outlined in Table 2). These differences in treatment, as well as information on therapy disruption or withdrawal, were not considered when pooling clinical outcomes between studies. If multiple dosing schedules were described, outcomes from one dosing regimen were chosen. For example, for one study that evaluated 2 dosing schedules of DEC—DEC-5 day, and DEC-10 day—only the DEC-10-day outcomes were included in this analysis, because this group had a greater number of patients and was consistent with the dosing schedule used in the other study of DEC included in the analysis [19].

**Table 2:** Summary of treatment dosages, frequency, and duration in the 12 studies evaluated in the meta-analysis

Study name (Other identifier)	Study type	Sample size	Tx group	Drug(s) and dosage(s)	Frequency	Duration of Tx
AZA-AML-001 [20] (NCT01074047)	RCT	241	HMA	AZA 75 mg/m^2^/d	7 d/28-d cycle	Minimum 6 cycles
CALGB 11,002 [19] (NCT01420926)	RCT	82	HMA	DEC 20 mg/m^2^/d	10 d/28-d cycle	4 cycles to achieve remission, 2 more if not achieved. Continuation therapy: same dosage, 5 d/28-d cycle. Maintenance therapy is same
Short 2019 [21]	RCT	28	HMA	DEC 5-d 20 mg/m^2^/d	5 consecutive d every 4–8 weeks	–
		43		DEC 10-d 20 mg/m^2^/d	10 consecutive d every 4–8 weeks	–
VIALE-A [6] (NCT02993523)	RCT	145	HMA	AZA 75 mg/m^2^/d	7 d/28-d cycle	–
		286	VEN + HMA	VEN 400 mg/d + AZA 75 mg/m^2^/d	Daily for 28 d; 7 d/28-d cycle	–
DiNardo 2018 [22] (NCT02203773)	Single arm	84	VEN + HMA	VEN 400 mg/d + AZA 75 mg/m^2^/d	–	–
		31		VEN 400 mg + DEC 20 mg/m^2^/d	–	–
DiNardo 2020 [23] (NCT03404193)	Single arm	37	VEN + HMA	VEN escalation over 3 d to 400 mg (100, 200, 400) + DEC 20 mg/m^2^/d	10 d /28-d cycle	Until remission. Remission: VEN given 1–21, instead of 1–28. Decrease to 14–10-7 depending on cytopenia
Kadia 2015 [24]	RO	293	IC/HMA	HDAC-based/HMA	–	–
Short 2020 [25] (NCT01786343)	RO	202	IC/HMA ± VEN	IDAC- or HDAC-based/HMA ± VEN	–	–
Lindsley 2019 [26] (NCT01696084)	RCT	156	IC	7 + 3 cytarabine 100 mg/m^2^/d + daunorubicin 60 mg/m^2^/d	1–7 d; 1–3 d	Second induction 5 + 2
Prochazka 2019 [13] (AML-HD98A; AML-HD98B; AMLSG-07–04)	RCT	98	IC	IC	–	–
Chiche 2021 [27]	RO	103	IC	CPX-351 (daunorubicin 44 mg/m^2^ + cytarabine 100 mg/m^2^)	d1 and d3	1 or 2 cycles
Desoutter 2014 [28]	RO	96	HMA	AZA 75 mg/m^2^/d	7 d/28-d cycle	4–6 cycles

*AZA* azacitidine, *d* day, *DEC* decitabine, *HDAC* high-dose cytarabine, *HMA* hypomethylating agent, *IC* intensive chemotherapy, *IDAC* intermediate-dose cytarabine, *RCT* randomized controlled trial, *RO* retrospective observational, *Tx* treatment, *VEN* venetoclax

### Outcome definitions

The response outcomes of interest were CR, which was defined according to International Working Group 2003 criteria as bone marrow blasts < 5%, platelets ≥ 100,000/µL, and neutrophils > 1000/µL [29]; CR with CRi, which was defined as CR with residual neutropenia (absolute neutrophil count < 1000 cells/µL) or thrombocytopenia (platelets < 100,000/µL); CR/CRi, achievement of either CR or CRi during the study period; CR with incomplete platelet recovery (CRp), achievement of complete remission that is accompanied by incomplete platelet recovery (platelets < 100,000/µL); morphologic leukemia-free state (MLFS), defined as bone marrow blasts < 5%, the absence of blasts with Auer rods, and the absence of extramedullary disease (no hematologic recovery required); and partial remission (PR), defined as all hematologic criteria of CR, a decrease of 5% to 25% in bone marrow blast percentage, and a decrease of pretreatment bone marrow blast percentage by ≥ 50%.

Survival outcomes consisted of OS—the length of time from the date of diagnosis or the date from the start of treatment to the death of the patient—and event-free survival (EFS)—the length of time after primary treatment the patient remained free of adverse outcomes, such as disease progression, local or distant recurrence, or death due to any cause.

Overall response rate (ORR) was defined as the achievement of any of the following: CR + CRi + CRp + MLFS + PR. Because certain studies reported ORRs that were defined differently, any differences in ORR study outcomes are noted. Duration of response (DoR) was the length of time the malignancy continued to respond to therapy without growing or spreading. Not all studies reported on all outcomes or events of interest; outcomes were pooled as appropriate with associated sample sizes reported.

### Quality assessments

Quality assessments were performed by 2 investigators working independently.

#### RCTs

The Risk of Bias 2 instrument, endorsed by the Cochrane Collaboration, was used to determine the validity of all included RCTs [30]. This instrument includes 5 domains of potential bias: arising from the randomization process, deviations from intended interventions, missing outcome data, measurement of the outcome, and selection of the reported result. Within each domain, investigators evaluated the risk of bias as low, some concerns, or high to provide an overall judgement about the risk of bias in the RCT.

Within the clinical context of this review, efficacy outcomes were well defined and objective within the classification criteria applied, and most studies were of open-label design (Additional File 2: Table S2).

#### Observational studies

The Newcastle–Ottawa scale was used to assess the quality of observational studies [31]. For each question, a response from a list is selected, with certain responses providing a star to the study. A greater number of stars is indicative of a lower risk of bias, whereas fewer stars indicate a higher risk of bias (Additional File 3: Table S3). Questions that are not applicable, such as those in the context of a single-arm trial, were denoted with “N/A.” For cohort studies, the scale is composed of 3 sections: selection, comparability, and outcome.

### Data analysis

Random-effects meta-analyses were conducted for dichotomous outcomes and helped account for between-study heterogeneity [32]. Median OS was synthesized with the median of medians method [33]. The primary analyses were performed only for patients with *TP53*m AML. A subgroup analysis based on study design (i.e., RCTs and prospective and retrospective observational studies) was also conducted whenever there were sufficient data. Data were maintained in Microsoft Excel 2016 workbooks. Using the packages of metafor [34], meta [32], and meta-median [33, 35], statistical analyses were conducted in R version 4.0.3 (2020-10-10; https://www.r-project.org/).

## Results

### Study selection

**Fig. 1:**
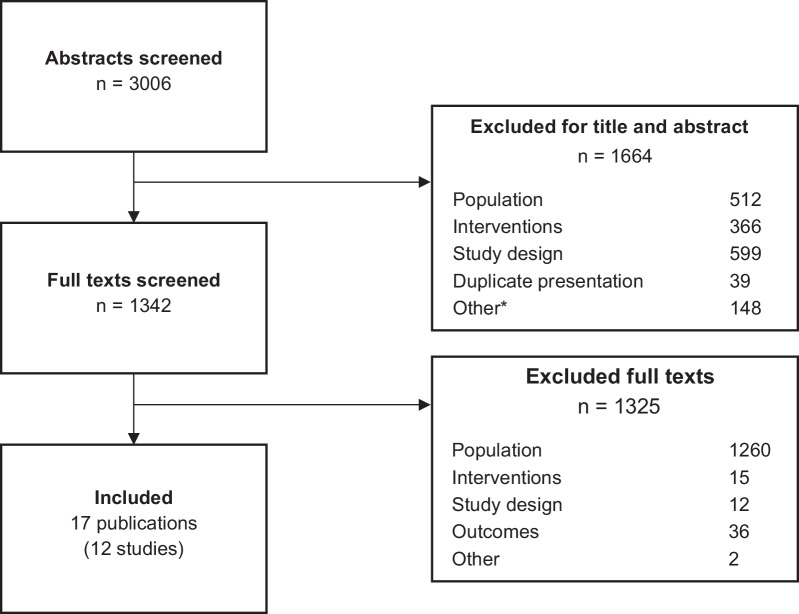
Study selection process. * “Other” reasons included commentary and opinion articles, protocols, review studies, lab science studies, and studies with no outcomes of interest

Results of the review process are summarized in Fig. 1. Systematic searches of EMBASE and MEDLINE identified 3006 abstracts, of which 512 were excluded for having different populations; 366, for not evaluating interventions of interest; 599, for ineligible study design; 39, for being duplicate publications; and 148, for other reasons. “Other” reasons for exclusion included type of article (i.e., commentary/opinion, protocols, review studies, and lab science studies) and studies with no outcomes of interest. Consequently, 1342 papers were reviewed in full-text, and 1325 of these were excluded for the following reasons: different populations than the one of interest (n = 1260), no evaluation of the interventions of interest (n = 15), ineligible study design (n = 12), no report of the outcomes of interest (n = 36), and other reasons (n = 2). Therefore, 17 publications representing 12 unique studies comprised the evidence base of this review and meta-analysis [6, 13, 19–28, 36–40].

Of the 12 studies identified, 6 were RCTs, 2 were single-arm clinical trials, and 4 were retrospective observational studies. In the clinical trials, the sample sizes ranged from 37 to 431, whereas in the retrospective studies, the sample sizes ranged from 96 to 293. IC was evaluated in 5 studies: 2 RCTs and 3 retrospective studies. Among the RCTs, cytarabine + daunorubicin and cytarabine, idarubicin, etoposide, or pegfilgrastim were evaluated. Among the retrospective studies, high-dose cytarabine (HDAC), an intermediate-dose cytarabine (IDAC)/HDAC-based regimen, and CPX-351 were evaluated. HMA was evaluated in 6 studies: 4 RCTs and 2 retrospective studies (3 used AZA, 2 used DEC, and 1 did not specify the HMA used). VEN + HMA was evaluated in 4 studies: 3 clinical trials and 1 retrospective study. Details of the regimens, doses, and number of patients enrolled are shown in Table 2; age, sex, and Eastern Cooperative Oncology Group (ECOG) status are shown in Table 3; and type of AML, cytogenetics, and bone marrow blasts are shown in Table 4.

**Table 3:** Age, sex, and ECOG status of patients in the 12 studies evaluated in the meta-analysis

Study name (other identifier)	Sample size	Age, median (range)	Tx	Male, n (%)	ECOG status, n (%)				
					0	1	2	3	4
AZA-AML-001 [20]	241	75 (6)^a^	AZA	139 (57.7)	54 (22.4)	132 (54.8)	55 (22.8)	0	0
CALGB 11,002 [19]	82	72 (61–92)	DEC	51 (62.2)	22 (26.8)	41 (50.0)	16 (19.5)	3 (3.7)	–
Short 2019 [21]	28	77 (70–80)^b^	DEC 5-day	–	18 (64.3)	–	10 (35.7)	–	–
	43	78 (69–82)^b^	DEC 10-day	–	30 (69.8)	–	13 (30.2)	–	–
VIALE-A [6]	145	76 (60–90)	AZA	87 (60.0)	81 (55.9)	–	64 (44.1)	–	–
	286	76 (49–91)	VEN + AZA	172 (60.1)	157 (54.9)	–	129 (45.1)	–	–
DiNardo 2018 [22]	84	75 (61–90)	VEN + AZA	51 (60.7)	14 (16.7)	44 (52.4)	24 (28.6)	2 (2.4)	–
	31	72 (65–86)	VEN + DEC	15 (48.4)	7 (22.6)	20 (64.5)	4 (12.9)	0	–
DiNardo 2020 [23]	37	74 (69–78)^b^	VEN + DEC	20 (54.1)	26 (70.3)	–	11 (29.7)	–	–
Kadia 2015 [24]	293	–	HDAC/HMA	–	–	–	–	–	–
Short 2020 [25]	202	70 (20–90)	IDAC- or HDAC-based/HMA ± VEN	–	–	–	–	–	–
Lindsley 2019 [26]	156	68 (4)^a^	7 + 3 cytarabine + daunorubicin	96 (61.5)	–	–	–	–	–
Prochazka 2019 [13]	98	57 (20–79)	IC	60 (61.2)	–	–	–	–	–
Chiche 2021 [27]	103	67 (20–83)	CPX-351 (daunorubicin + cytarabine)	54 (52.4)	–	–	–	–	–
Desoutter 2014 [28]	96	73 (44–88)	AZA	–	–	–	–	–	–

*AZA* azacitidine, *DEC* decitabine, *ECOG* Eastern Cooperative Oncology Group, *HDAC* high-dose cytarabine, *HMA* hypomethylating agent, *IC* intensive chemotherapy, *IDAC* intermediate-dose cytarabine, *IQR* interquartile range, *Tx* treatment, *VEN* venetoclax

^a^Mean (± SD)

^b^Median (± IQR)

**Table 4:** Type of AML, cytogenetic risk category, and BMBC in patients in the 12 studies evaluated in the meta-analysis

Study name (Other identifier)	Sample size	Tx	Type of AML			Cytogenetic risk category			BMBC, median (range)
			De novo, n (%)	Secondary, n (%)	Therapy-related, n (%)	Poor, n (%)	Intermediate, n (%)	No mitosis, n (%)	
AZA-AML-001 [20]	241	AZA	–	–	–	–	–	–	66.6 (24.7%)^a^
CALGB 11,002 [19]	82	DEC	–	–	–	–	–	–	–
Short 2019 [21]	28	DEC 5-day	–	13 (46.4)	–	–	–	–	40 (29–68)^b^
	43	DEC 10-day	–	18 (41.9)	–	–	–	–	46 (25–64)^b^
VIALE-A [6]	145	AZA	110 (75.9)	26 (17.9)	9 (6.2)	56 (38.6)	89 (61.4)	–	–
	286	VEN + AZA	214 (74.8)	46 (16.1)	26 (9.1)	104 (36.4)	182 (63.6)	–	–
DiNardo 2018 [22]	84	VEN + AZA	–	–	–	33 (39.3)	50 (59.5)	1 (1.2)	–
	31	VEN + DEC	–	–	–	15 (48.4)	16 (51.6)	–	–
DiNardo 2020 [23]	37	VEN + DEC	–	–	–	–	–	–	26 (–)^a^
Kadia 2015 [24]	293	HDAC/HMA	–	–	–	–	–	–	–
Short 2020 [25]	202	IDAC- or HDAC-based/HMA ± VEN	–	30 (14.9)	52 (25.7)	–	–	–	32 (3–97)
Lindsley 2019 [26]	156	7 + 3 cytarabine + daunorubicin	–	–	–	–	–	–	–
Prochazka 2019 [13]	98	IC	82 (83.7)	4 (4.1)	12 (12.2)	2 (2.0)	11 (11.2)	71 (72.4)	60 (14–100)
Chiche 2021 [27]	103	CPX-351 (daunorubicin + cytarabine)	–	–	27 (26.2)	–	–	–	–
Desoutter 2014 [28]	96	AZA	–	53 (55.2)	14 (14.6)	61 (63.5)	21 (21.9)	14 (14.6)	15 (0–95)

*AML* acute myeloid leukemia, *AZA* azacitidine, *BMBC* bone marrow blast count, *DEC* decitabine, *HDAC* high-dose cytarabine, *HMA* hypomethylating agent, *IC* intensive chemotherapy, *IDAC* intermediate-dose cytarabine, *Tx* treatment, *VEN* venetoclax

^a^Mean (± SD)

^b^Median (± IQR)

### Outcome results

#### CR

**Fig. 2:**
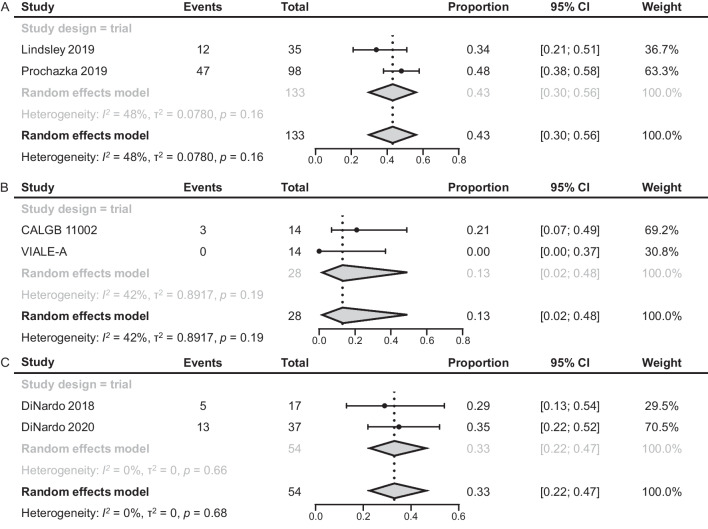
CR in patients with *TP53*m AML treated with IC (**A**), HMA (**B**), and VEN + HMA (**C**). AML, acute myeloid leukemia; CI confidence interval; CR, complete remission; HMA, hypomethylating agent; IC, intensive chemotherapy; *TP53*m, *TP53*-mutated; VEN, venetoclax

Two RCTs (N = 133) yielded CR rates of 34% (95% confidence interval [CI], 21–51%; n = 35) and 48% (38–58%; n = 98) for patients with *TP53*m AML treated with IC (Fig. 2A) [13, 26]. The pooled rate of CR after treatment with IC was 43% (30–56%).

When HMA was used to treat *TP53*m AML, the reported CR rates in 2 RCTs (N = 28) were 21% (95% CI 7–49%; n = 14) [19] and 0 (0–37%; n = 14) [6], and the pooled CR rate was 13% (2–48%; Fig. 2B).

Rates of CR in the 2 RCTs (N = 54) that investigated the use of VEN + HMA were 29% (95% CI 13–54%; n = 17) and 35% (22–55%; n = 37), and the pooled CR rate was 33% (22–47%; Fig. 2C) [22, 23].

#### CRi

**Fig. 3:**
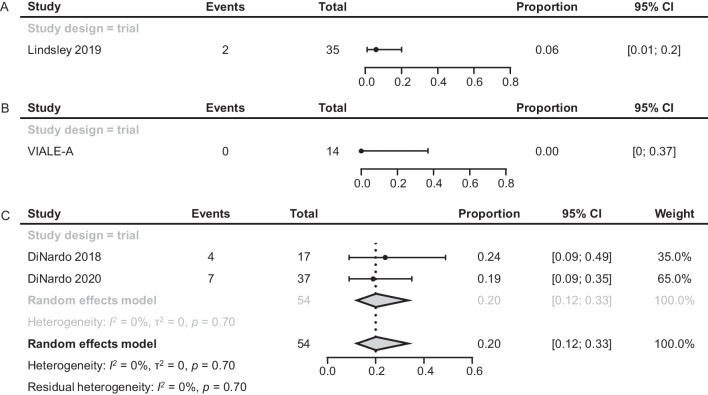
CRi of patients with *TP53*m AML treated with IC (**A**), HMA (**B**), or VEN + HMA (**C**). AML, acute myeloid leukemia; CI confidence interval; CR, complete remission; CRi, CR with incomplete hematologic recovery; HMA, hypomethylating agent; IC, intensive chemotherapy; *TP53*m, *TP53*-mutated; VEN, venetoclax

Results for CRi of patients treated with IC or HMA were limited in the literature. In 1 study of patients treated with IC (N = 35), 2 patients achieved CRi (6%; 95% CI 1–20%; Fig. 3A) [26].

One RCT reported a CRi rate of 0 (95% CI 0–37%; Fig. 3B) for *TP53*m AML treated with HMA alone [6].

Two studies of VEN + HMA (N = 54) reported CRi rates: 24% (95% CI 9–49%; n = 17) and 19% (9–35%; n = 37; Fig. 3C) [22, 23]. The pooled rate was 20% (12–33%).

#### CR/CRi

**Fig. 4:**
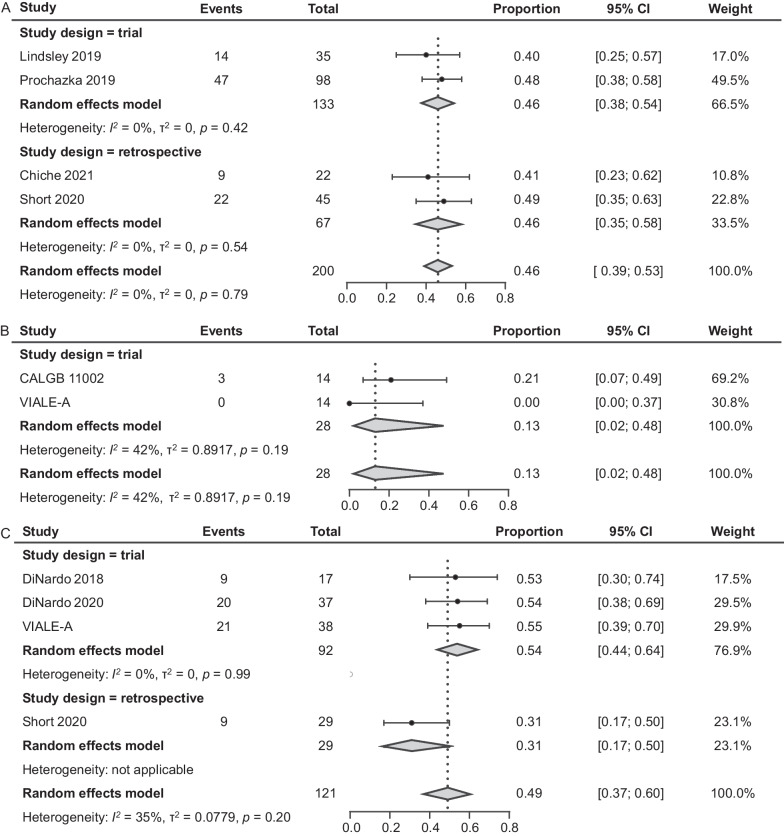
CR/CRi of patients with *TP53*m AML treated with IC (**A**), HMA (**B**), or VEN + HMA (**C**). AML, acute myeloid leukemia; CI confidence interval; CR, complete remission; CRi, CR with incomplete hematologic recovery; HMA, hypomethylating agent; IC, intensive chemotherapy; *TP53*m, *TP53*-mutated; VEN, venetoclax

Four studies of IC reported CR/CRi rates [13, 25–27]. For the 2 RCTs (N = 133), the CR/CRi rates were 40% (95% CI 25–57%; n = 35) and 48% (38–58%; n = 98), and the pooled rate for these RCTs was 46% (38–54%; Fig. 4A) [13, 26]. Two retrospective studies of IC yielded similar rates of CR/CRi: 41% (23–62%; n = 22) and 49% (35–63%; n = 45); the pooled rate for these retrospective studies was 46% (35–58%) [25, 27]. The pooled CR/CRi rate across all 4 studies was 46% (39–53%).

CR/CRi rates were low in the 2 RCTs (N = 28) that investigated HMA as treatment of *TP53*m AML [6, 19]. In one study, the CR/CRi rate was reported as 0 (95% CI 0–37%; n = 14), and in the other study, the rate was 21% (7–49%; n = 14; Fig. 4B). The pooled CR/CRi rate was 13% (2–48%).

For VEN + HMA, the CR/CRi rates in 3 clinical trials (N = 92) ranged from 53% (95% CI 30–72%; n = 17) to 55% (39–70%; n = 38), resulting in a pooled CR/CRi rate of 54% (44–64%; Fig. 4C) [6, 22, 23]. In the single retrospective study, the reported CR/CRi rate was 31% (17–50%; n = 29) [25]. Across the 4 studies, the pooled CR/CRi rate was 49% (37–60%).

#### Median OS

**Fig. 5:**
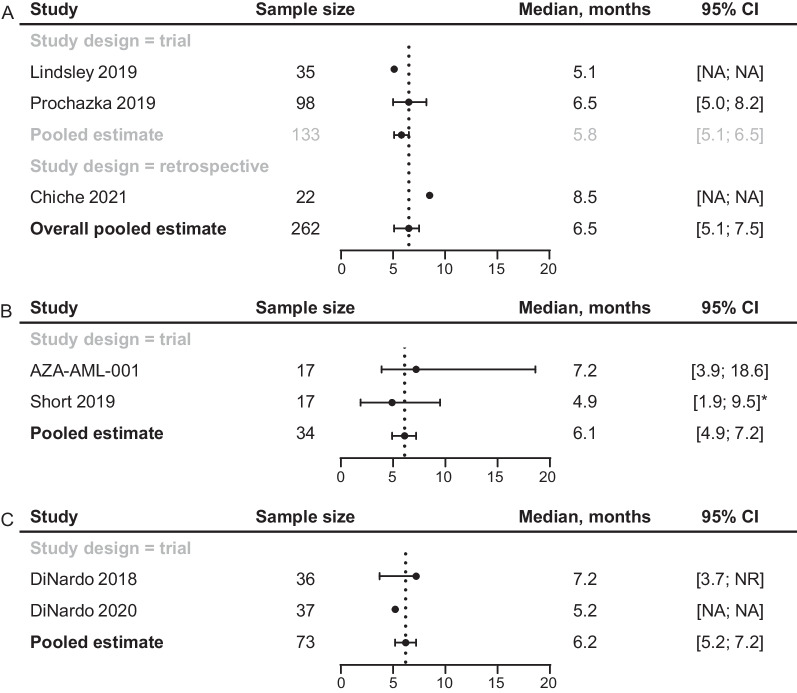
Median OS of patients with *TP53*m AML treated with IC (**A**), HMA (**B**), or VEN + HMA (**C**). *IQR was reported. AML, acute myeloid leukemia; CI confidence interval; HMA, hypomethylating agent; IC, intensive chemotherapy; IQR, interquartile range; NA, not reported; NR, not reached; OS, overall survival; *TP53*m, *TP53*-mutated; VEN, venetoclax

The median OS estimates for the 3 types of treatment evaluated for *TP53*m AML were comparable (Fig. 5). The median OS in 2 RCTs (N = 133) that evaluated IC was 5.1 months (95% CI not reported; n = 35) and 6.5 months (5.0–8.2; n = 98), and the pooled median OS estimate for RCTs was 5.8 months (5.1–6.5) [13, 26]. The median OS estimate was higher in the single retrospective study of IC: 8.5 months (95% CI not reported; n = 22) [27]. Across the 3 studies, the pooled median OS was 6.5 months (5.1–7.5).

In 2 RCTs (N = 34) of HMA, the median OS estimates were 4.9 months (interquartile range [IQR], 1.9–9.5; n = 17) and 7.2 months (95% CI 3.9–18.6; n = 17) [20, 21]. The pooled median OS across all study designs was 6.1 months (95% CI 4.9–7.2).

Similarly, the median OS estimates for 2 clinical trials (N = 73) of VEN + HMA were 5.2 months (95% CI not reported; n = 37) and 7.2 months (3.7, not reached; n = 36) [22, 23]. The pooled median OS estimate for the VEN + HMA studies was 6.2 months (5.2–7.2).

#### EFS

EFS estimates were reported from 2 RCTs (N = 133) of IC and were 1.6 months (95% CI not reported; n = 35) and 5.7 months (4.3–7.4; n = 98) [13, 26]. When estimates were pooled, the result was 3.7 months (1.6–5.7).

The outcome of EFS was not reported in any studies of patients treated with HMA or VEN + HMA.

#### ORR

Single studies of IC, HMA, and VEN + HMA reported ORR using different definitions across studies, and no ORR value exceeded 65%. In a retrospective study of IC (n = 22), the ORR—defined as CR or CRi—was 41% [27]. A similar ORR of 47% was reported from an RCT of HMA (n = 17); in this study, the ORR was based on CR, CRp, CRi, or PR [21]. A trial of VEN + HMA (n = 37) yielded an ORR of 65%; ORR was not defined in this study [37].

#### DoR

The estimates of DoR were 7 months or less for any treatment arm reported across all included studies with DoR data available [22, 23, 26]. A single RCT of IC (n = 35) found a median DoR of 3.5 months, whereas no identified studies of HMA reported a median DoR [26].

The highest estimates of median DoR were seen in 2 clinical trials of VEN + HMA (N = 54): 6.5 months (95% CI 1.9–17.3; n = 17) and 3.5 months (95% CI 2.1–16.6; n = 37), with a pooled DoR of 5.0 months (95% CI 3.5–6.5) [22, 23]. Patients who achieved CR had a median DoR of 7.0 months, whereas patients who achieved CRi had a median DoR of 2.5 months. The study reported by DiNardo, et al. (2020) [23], with a 3.5-month DoR for VEN + HMA, did not censor patients at stem cell transplant; this information was not available for the other studies.

#### CRp, MLFS, and PR

The 12 studies identified in our review did not report CRp, MLFS, and PR; therefore, these outcomes were not evaluated in our analysis.

## Discussion

This comprehensive and most recent systematic review and meta-analysis was undertaken to evaluate outcomes associated with IC, HMA, and VEN + HMA treatments of newly diagnosed, treatment-naïve patients with *TP53*m AML. Findings from this study confirm that patients with *TP53*m AML experience poor outcomes regardless of the type of therapy received. CR rates ranged between 13 and 43% for treatments across studies in the pooled data analysis, whereas CR/CRi rates tended to be between 13 and 49% among the studies that reported such results. CR and CR/CRi rates were better among IC- and VEN + HMA–treated patients compared with HMA alone, but they were still low in comparison to the CR rate of 85% previously reported in *TP53* wild-type AML [13, 37, 41]. IC reported the highest pooled CR/CRi and CR rates (46% and 43%, respectively). However, this may represent a selection bias wherein younger and fitter patients who are often more likely to progress to allogeneic stem cell transplantation were historically selected for IC, even in the *TP53*m setting. With VEN + HMA, the pooled CR rate was only 33% but was higher than the CR rate of 21% for HMA alone.

Median OS estimates for each treatment type were uniformly low, ranging from 6.1 months in the HMA cohort to 6.2 months in the HMA + VEN cohort to 6.5 months for IC. Despite the better CR and CR/CRi rates among IC- and VEN + HMA–treated patients, the pooled median OS of each was similar to that of HMA alone, and all were < 7.0 months, suggesting that improved treatment responses with IC and VEN + HMA did not translate to improved OS.

p53 is key to apoptosis resulting from cytotoxic chemotherapy; therefore, mutated p53 can result in resistance to DNA-damaging chemotherapies that are used to treat AML [7]. Additionally, preclinical studies have shown that p53 loss-of-function in isogenic human AML cell lines results in resistance to HMA treatment with or without VEN [42]. This potentially contributes to the lower rates of CR and CR/CRi and the reduced OS observed in this extremely difficult-to-treat population. Furthermore, *TP53*m patients tend to have greater degrees of myelosuppression and higher early mortality, with reported early (60-day) mortality rates as high as 26% in a contemporary study at MD Anderson Cancer Center that treated *TP53*m patients with HMA + VEN, compared with 60-day mortality rates of 4% in non-*TP53*m patients treated with HMA + VEN at the same institution [14]. Novel therapies that directly target pathways other than those involving p53 are being aggressively evaluated to improve clinical outcomes of patients with newly diagnosed, treatment-naïve *TP53*m AML [7].

Possible new therapies for *TP53*m AML include immunotherapy, such as bispecific antibodies, chimeric receptor antigen (CAR) T-cell therapy, and monoclonal antibodies [7]. Immunotherapies that facilitate effector T-cell responses have been used widely to treat other types of malignancies and are now being investigated as treatments for AML [43]. These adaptive immune checkpoint inhibitors alone or in combination with induction chemotherapy or HMA are being evaluated in various subtypes of AML including in *TP53*m AML [44]. Early studies of bispecific antibodies and CAR T-cell therapy have suggested each therapeutic modality has promise, but a disadvantage of both approaches is the need to target specific antigens, which is challenging in AML, as antigen expression on AML cells is not as specifically or differentially expressed compared to other hematologic malignancy types [7].

Magrolimab is a monoclonal antibody specific for CD47, a leukemic stem cell marker and the ligand for a macrophage immune checkpoint molecule called signal regulatory protein alpha (SIRPα) [45, 46]. By binding SIRPα, CD47 triggers a signal transduction cascade that results in a “don’t eat me” signal communicated from the malignant cell to the macrophage [45, 46]. Phase 1b/2 studies are investigating the efficacy and safety of magrolimab in combination with AZA, which synergizes with magrolimab by inducing the “eat me” signals on leukemic cells, and in combination with AZA + VEN in AML [45]. In the Phase 1b study of magrolimab in combination with AZA, ORR among patients with *TP53*m AML (n = 72) was 48.6% (33.3% CR, 8.3% CRi, and 5.6% PR), and median OS was 10.8 months [47]. This median OS is encouraging when reviewed in context of the OS with IC, VEN + HMA, or HMA alone as shown in this paper, with median OS ranging from 6.1 to 6.5 months with these modalities. A Phase 3 trial (NCT04778397) comparing the efficacy and safety of magrolimab + AZA with that of VEN + AZA or IC in adult patients with newly diagnosed *TP53*m AML is ongoing [48].

Another compound in development for *TP53m* AML is eprenetapopt, a novel, first-in-class, small molecule that induces *TP53*m cell apoptosis. Eprenetapopt in combination with AZA showed promise in a Phase 1b/2 trial of *TP53*m myelodysplastic syndrome and AML patients [49, 50]. As a result, a Phase 1 trial of eprenetapopt + AZA + VEN was initiated. This study reported encouraging early efficacy data and is ongoing [51].

A regimen of 10-day DEC showed favorable clinical responses (including CR/CRi) among patients with *TP53*m compared to patients with wild-type *TP53* in a single institution trial [52]; however, this was not reproduced in a randomized Phase 2 study comparing 5-day versus 10-day DEC [21].

This systematic review and meta-analysis provides insight and establishes clinical outcome benchmarks using contemporary literature and therapies in patients with *TP53*m AML receiving different types of therapies. While patient and treatment selection criteria limited the number of articles included in this study, strict inclusion and exclusion criteria were added to optimize the validity of the findings. The results of the full-text screening were cross-referenced with published systematic literature reviews on similar topics to ensure the inclusion of all relevant publications. The scope of the review was broad, encompassing RCTs, nonrandomized or single-arm trials, and prospective or retrospective observational studies. The Risk of Bias 2 tool and the Newcastle–Ottawa scale were used to assess the strength of evidence available for each outcome in the context of AML research. However, a limitation of this analysis, as with similar systematic reviews and meta-analyses, is that the analyses for all outcomes were based on the pooling of proportions from each intervention group, rather than comparative evidence. Due to the limited number of available studies and the lack of details about outcomes of specific intensive or nonintensive regimens, we were unable to compare outcomes between individual treatment regimens. This represents an important topic for future study of *TP53*m AML, as it is possible that outcomes could differ based on the specific intensive or nonintensive regimen applied. Additionally, we were unable to make reliable comparisons between treatment regimens within age subgroups owing to the greater likelihood that older patients received HMA or VEN + HMA over IC. It is also important to note that each study enrolled different patient populations using different eligibility criteria, and each study was conducted over different time periods. These factors most likely impacted both response and survival outcomes. Consequently, it must be clearly highlighted that it was not the intent of this analysis to draw conclusions or to infer the relative effectiveness of these interventions compared to each other or to other treatments. Furthermore, this analysis did not explore methods of controlling for heterogeneity other than stratification through study design.

## Conclusions

Estimates of CR, median OS, and other measures of efficacy were low across treatments, including IC, HMA, and VEN + HMA, for patients with newly diagnosed, treatment-naïve *TP53*m AML. Though adding VEN to HMA improved CR and CR/CRi rates compared with HMA alone, median OS was not prolonged. Median OS remained dismal at 6.1, 6.2, and 6.5 months for HMA alone, VEN + HMA, and IC, respectively, highlighting the dire unmet need in this population of myeloid malignancies. Findings from this study point to a substantial need for new therapies that can effectively treat newly diagnosed, treatment-naïve *TP53*m AML and improve outcomes.

## Supplementary Information

**Additional file 1: Table S1.** Strategy for searches of MEDLINE and EMBASE databases on May 20, 2021.

**Additional file 2: Table S2**. Risk of Bias 2 assessment for RCTs.

**Additional file 3: Table S3.** Newcastle-Ottawa scale assessment for observational studies.

## Data Availability

Data will be shared on request for research purposes dependent on the nature of the request, the merit of the proposed research, the availability of the data, and its intended use. The full data sharing policy for Gilead Sciences, Inc., can be found at https://www.gilead.com/science-and-medicine/research/clinical-trials-transparency-and-data-sharing-policy.

## Abbreviations

AML: Acute myeloid leukemia
AZA: Azacitidine
CAR: Chimeric receptor antigen
CR: Complete remission
CRi: Complete remission with incomplete hematologic recovery
CRp: Complete remission with incomplete platelet recovery
DEC: Decitabine
DoR: Duration of response
EFS: Event-free survival
HMA: Hypomethylating agents
IC: Intensive chemotherapy
MLFS: Morphologic leukemia-free state
ORR: Overall response rate
OS: Overall survival
PICOS: Population, interventions, comparisons, outcomes, and study design
PR: Partial remission
PRISMA: Preferred reporting items for systematic reviews and meta-analyses
RCT: Randomized controlled trial
SIRPα: Signal regulatory protein alpha
*TP53*m: Mutated *TP53*
VEN: Venetoclax
