# Supplementary material for: Treatment outcomes for newly diagnosed, treatment-naïve TP53-mutated acute myeloid leukemia: a systematic review and meta-analysis
Source: J Hematol Oncol. 2023 Mar 6;16:19. doi: 10.1186/s13045-023-01417-5 (PMC9990239; doi:10.1186/s13045-023-01417-5)
Supplement: Supplementary file 1 — Additional file 1: Table S1. Strategy for searches of MEDLINE and EMBASE databases on May 20, 2021. [file 13045_2023_1417_MOESM1_ESM.docx]

Table S1. Strategy for searches of MEDLINE and EMBASE databases on May 20, 2021

|  | **Search number** | **Query** | **Results** |
| --- | --- | --- | --- |
| **Population** | 1 | acute myeloid leukemia.mp. | 106,663 |
|  | 2 | AML.mp. | 103,475 |
|  | 3 | 1 or 2 | 142,854 |
|  | 4 | ((first* adj2 line) or untreat* or (treatment adj2 naive) or (new* adj2 diagnos*)).ti,ab. | 902,579 |
|  | **5** | **3 and 4** | **12,617** |
| **Interventions** | 6 | (intens* or induct*).mp. | 3,570,935 |
|  | 7 | chemo*.mp. | 2,238,281 |
|  | 8 | 6 and 7 | 252,537 |
|  | 9 | (Cytarabine or cytosine arabinoside or ara-C).mp. | 86,032 |
|  | 10 | (Cladribine or Leustatin).mp. | 9,734 |
|  | 11 | (Fludarabine or Fludara).mp. | 36,886 |
|  | 12 | (MEC or Mitoxantrone).mp. | 46,337 |
|  | 13 | (((daunorubicin or daunomycin or rubidomycin hydrochloride or Cerubidine) and (cytarabine or cytosine arabinoside or ara-C or Arabinosylcytosine or Cytosar-U)) or Vyxeos).mp. | 18,978 |
|  | 14 | or/8-13 | 385,495 |
|  | 15 | hypomethyl*.mp. | 25,645 |
|  | 16 | (Decitabine or Dacogen).mp. | 12,073 |
|  | 17 | (Azacitidine or Azacytidine or Vidaza).mp. | 24,775 |
|  | **18** | **or/14-17** | **428,819** |
| **Study design** | 19 | (Randomized Controlled Trial or Controlled Clinical Trial or Pragmatic Clinical Trial or Equivalence Trial or Clinical Trial, Phase III).pt. | 624,162 |
|  | 20 | Randomized Controlled Trial/ | 1,187,553 |
|  | 21 | exp Randomized Controlled Trials as Topic/ | 350,124 |
|  | 22 | "Randomized Controlled Trial (topic)"/ | 202,763 |
|  | 23 | Controlled Clinical Trial/ | 557,431 |
|  | 24 | exp Controlled Clinical Trials as Topic/ | 363,638 |
|  | 25 | "Controlled Clinical Trial (topic)"/ | 11,584 |
|  | 26 | Randomization/ | 196,079 |
|  | 27 | Random Allocation/ | 192,236 |
|  | 28 | Double-Blind Method/ | 323,518 |
|  | 29 | Double Blind Procedure/ | 183,841 |
|  | 30 | Double-Blind Studies/ | 306,832 |
|  | 31 | Single-Blind Method/ | 70,703 |
|  | 32 | Single Blind Procedure/ | 42,545 |
|  | 33 | Single-Blind Studies/ | 72,740 |
|  | 34 | Placebos/ | 345,200 |
|  | 35 | Placebo/ | 366,084 |
|  | 36 | Control Groups/ | 111,707 |
|  | 37 | Control Group/ | 111,707 |
|  | 38 | (random* or sham or placebo*).ti,ab,hw,kf,kw. | 3,779,267 |
|  | 39 | ((singl* or doubl*) adj (blind* or dumm* or mask*)).ti,ab,hw,kf,kw. | 567,867 |
|  | 40 | ((tripl* or trebl*) adj (blind* or dumm* or mask*)).ti,ab,hw,kf,kw. | 2,744 |
|  | 41 | (control* adj3 (study or studies or trial* or group*)).ti,ab,kf,kw. | 2,521,630 |
|  | 42 | (Nonrandom* or non random* or non-random* or quasi-random* or quasirandom*).ti,ab,hw,kf,kw. | 106,485 |
|  | 43 | allocated.ti,ab,hw. | 162,924 |
|  | 44 | ((open label or open-label) adj5 (study or studies or trial*)).ti,ab,hw,kf,kw. | 107,108 |
|  | 45 | ((equivalence or superiority or non-inferiority or noninferiority) adj3 (study or studies or trial*)).ti,ab,hw,kf,kw. | 23,363 |
|  | 46 | (pragmatic study or pragmatic studies).ti,ab,hw,kf,kw. | 1,148 |
|  | 47 | ((pragmatic or practical) adj3 trial*).ti,ab,hw,kf,kw. | 12,282 |
|  | 48 | ((quasiexperimental or quasi-experimental) adj3 (study or studies or trial*)).ti,ab,hw,kf,kw. | 23,478 |
|  | 49 | (phase adj3 (III or "3") adj3 (study or studies or trial*)).ti,hw,kf,kw. | 131,058 |
|  | **50** | **or/19-49** | **5,539,146** |
|  | 51 | observation*.mp. | 2,188,936 |
|  | 52 | registry.mp. | 352,353 |
|  | 53 | cohort studies/ | 852,232 |
|  | 54 | longitudinal studies/ | 280,224 |
|  | 55 | follow-up studies/ | 1,882,296 |
|  | 56 | prospective studies/ | 1,152,343 |
|  | 57 | retrospective studies/ | 1,691,903 |
|  | 58 | cohort.ti,ab. | 1,628,972 |
|  | 59 | longitudinal.ti,ab. | 624,600 |
|  | 60 | prospective.ti,ab. | 1,533,703 |
|  | 61 | retrospective.ti,ab. | 1,570,085 |
|  | **62** | **or/51-61** | **8,569,474** |
| **All study design** | **63** | **50 or 62** | **12,696,590** |
| **Base strategy** | 64 | 5 and 18 and 63 | 4,197 |
| **Exclusion terms** | 65 | (addresses or bibliography or case report or comment or editorial or guideline or in vitro or letter or news).pt. | 4,030,005 |
|  | 66 | case report/ or case reports/ | 4,788,485 |
|  | 67 | 65 or 66 | 8,375,527 |
|  | 68 | 64 not 67 | 4,026 |
| **Limit to human** | 69 | Limit 68 to humans | 3,761 |
| **Limit to English** | 70 | Limit 69 to English language | 3,652 |
| **Deduplicate** | **71** | **Remove duplicates from 70** | **3,006** |
