# Supplementary material for: Treatment outcomes for newly diagnosed, treatment-naïve TP53-mutated acute myeloid leukemia: a systematic review and meta-analysis
Source: J Hematol Oncol. 2023 Mar 6;16:19. doi: 10.1186/s13045-023-01417-5 (PMC9990239; doi:10.1186/s13045-023-01417-5)
Supplement: Supplementary file 2 — Additional file 2: Table S2. Risk of Bias 2 assessment for RCTs. [file 13045_2023_1417_MOESM2_ESM.docx]

Table S2. Risk of Bias 2 assessment for RCTs

| **Study name** | **Outcome available** | **Bias from randomization process** | **Bias from deviations from intended interventions** | **Bias from missing outcome data** | **Bias from measurement of outcome** | **Bias from selection of reported result** | **Overall bias** |
| --- | --- | --- | --- | --- | --- | --- | --- |
| **CR** | | | | | | | |
| AZA-AML-001 | No | ― | ― | ― | ― | ― | ― |
| CALGB 11002 | Yes | Low risk | Low risk | Low risk | Low risk | Low risk | Low risk |
| Short 2019 | No | ― | ― | ― | ― | ― | ― |
| VIALE-A | Yes | Low risk | Low risk | Low risk | Low risk | Low risk | Low risk |
| Lindsley 2019 | Yes | Low risk | Low risk | Low risk | Low risk | Low risk | Low risk |
| Prochazka 2019 | Yes | Low risk | Low risk | Low risk | Low risk | Low risk | Low risk |
| **CRi** | | | | | | | |
| AZA-AML-001 | No | ― | ― | ― | ― | ― | ― |
| CALGB 11002 | No | ― | ― | ― | ― | ― | ― |
| Short 2019 | No | ― | ― | ― | ― | ― | ― |
| VIALE-A | Yes | Low risk | Low risk | Low risk | Low risk | Low risk | Low risk |
| Lindsley 2019 | Yes | Low risk | Low risk | Low risk | Low risk | Low risk | Low risk |
| Prochazka 2019 | No | ― | ― | ― | ― | ― | ― |
| **CR/CRi** | | | | | | | |
| AZA-AML-001 | No | ― | ― | ― | ― | ― | ― |
| CALGB 11002 | Yes | Low risk | Low risk | Low risk | Low risk | Low risk | Low risk |
| Short 2019 | No | ― | ― | ― | ― | ― | ― |
| VIALE-A | Yes | Low risk | Low risk | Low risk | Low risk | Low risk | Low risk |
| Lindsley 2019 | Yes | Low risk | Low risk | Low risk | Low risk | Low risk | Low risk |
| Prochazka 2019 | Yes | Low risk | Low risk | Low risk | Low risk | Low risk | Low risk |
| **OS** | | | | | | | |
| AZA-AML-001 | Yes | Low risk | Low risk | Low risk | Low risk | Low risk | Low risk |
| CALGB 11002 | No | ― | ― | ― | ― | ― | ― |
| Short 2019 | Yes | Low risk | Low risk | Low risk | Low risk | Low risk | Low risk |
| VIALE-A | No | ― | ― | ― | ― | ― | ― |
| Lindsley 2019 | Yes | Low risk | Low risk | Some concerns | Low risk | Low risk | Some concerns |
| Prochazka 2019 | Yes | Low risk | Low risk | Some concerns | Low risk | Low risk | Some concerns |
| **EFS** | | | | | | | |
| AZA-AML-001 | No | ― | ― | ― | ― | ― | ― |
| CALGB 11002 | No | ― | ― | ― | ― | ― | ― |
| Short 2019 | No | ― | ― | ― | ― | ― | ― |
| VIALE-A | No | ― | ― | ― | ― | ― | ― |
| Lindsley 2019 | Yes | Low risk | Low risk | Some concerns | Low risk | Low risk | Some concerns |
| Prochazka 2019 | Yes | Low risk | Low risk | Some concerns | Low risk | Low risk | Some concerns |
| **ORR** | | | | | | | |
| AZA-AML-001 | No | ― | ― | ― | ― | ― | ― |
| CALGB 11002 | No | ― | ― | ― | ― | ― | ― |
| Short 2019 | Yes | Low risk | Low risk | Low risk | Low risk | Low risk | Low risk |
| VIALE-A | No | ― | ― | ― | ― | ― | ― |
| Lindsley 2019 | No | ― | ― | ― | ― | ― | ― |
| Prochazka 2019 | No | ― | ― | ― | ― | ― | ― |
| **DoR** | | | | | | | |
| AZA-AML-001 | No | ― | ― | ― | ― | ― | ― |
| CALGB 11002 | No | ― | ― | ― | ― | ― | ― |
| Short 2019 | No | ― | ― | ― | ― | ― | ― |
| VIALE-A | No | ― | ― | ― | ― | ― | ― |
| Lindsley 2019 | Yes | Low risk | Low risk | Some concerns | Low risk | Low risk | Some concerns |
| Prochazka 2019 | No | ― | ― | ― | ― | ― | ― |

CR, complete response; CRi, complete response with incomplete hematologic recovery; DoR, duration of response; EFS, event-free survival; ORR, overall response rate; OS, overall survival; RCT, randomized controlled trial.
