# Supplementary material for: Treatment outcomes for newly diagnosed, treatment-naïve TP53-mutated acute myeloid leukemia: a systematic review and meta-analysis
Source: J Hematol Oncol. 2023 Mar 6;16:19. doi: 10.1186/s13045-023-01417-5 (PMC9990239; doi:10.1186/s13045-023-01417-5)
Supplement: Supplementary file 3 — Additional file 3: Table S3. Newcastle-Ottawa scale assessment for observational studies. [file 13045_2023_1417_MOESM3_ESM.docx]

Table S3. Newcastle-Ottawa scale assessment for observational studies

| **Study name** | **Selection** | **Comparability** | **Outcome** |
| --- | --- | --- | --- |
| Kadia 2015 | **** | N/A | ** |
| Short 2020 | **** | N/A | ** |
| Chiche 2021 | ** | N/A | ** |
| Desoutter 2014 | **** | N/A | ** |

N/A, not applicable.
